# Dialysis for paediatric acute kidney injury in Cape Town, South Africa

**DOI:** 10.1007/s00467-024-06399-1

**Published:** 2024-05-11

**Authors:** Mignon I. McCulloch, Valerie A. Luyckx, Brenda Morrow, Peter Nourse, Ashton Coetzee, Deveshni Reddy, Christel Du Buisson, Jonathan Buckley, Ilana Webber, Alp Numanoglu, Gina Sinclair, Candice Nelson, Shamiel Salie, Kirsten Reichmuth, Andrew C. Argent

**Affiliations:** 1 Red Cross War Memorial Children’s Hospital (RCWMCH), Rondebosch, Cape Town, South Africa; 2 University of Cape Town, Cape Town, South Africa; 3 Tygerberg Children’s Hospital, University of Stellenbosch, Stellenbosch, South Africa

**Keywords:** Acute kidney injury, Peritoneal dialysis, Extracorporeal dialysis, Neonate: Infant, Child

## Abstract

**Background:**

Dialysis is lifesaving for acute kidney injury (AKI), but access is poor in less resourced settings. A “peritoneal dialysis (PD) first” policy for paediatric AKI is more feasible than haemodialysis in low-resource settings.

**Methods:**

Retrospective review of modalities and outcomes of children dialysed acutely at Red Cross War Memorial Children’s Hospital between 1998 and 2020.

**Results:**

Of the 593 children with AKI who received dialysis, 463 (78.1%) received PD first. Median age was 9.0 (range 0.03–219.3; IQR 13.0–69.6) months; 57.6% were < 1 year old. Weights ranged from 0.9 to 2.0 kg (median 7.0 kg, IQR 3.0–16.0 kg); 38.6% were < 5 kg. PD was used more in younger children compared to extracorporeal dialysis (ECD), with median ages 6.4 (IQR 0.9–30.4) vs. 73.9 (IQR 17.5–113.9) months, respectively (*p* = 0.001). PD was performed with Seldinger soft catheters (*n* = 480/578, 83%), predominantly inserted by paediatricians at the bedside (*n* = 412/490, 84.1%). Complications occurred in 99/560 (17.7%) children receiving PD. Overall, 314/542 (57.8%) children survived. Survival was significantly lower in neonates (< 1 month old, 47.5%) and infants (1–12 months old, 49.2%) compared with older children (> 1 year old, 70.4%, *p* < 0.0001). Survival was superior in the ECD (75.4%) than in the PD group (55.6%, *p* = 0.002).

**Conclusions:**

“PD First for Paediatric AKI” is a valuable therapeutic approach for children with AKI. It is feasible in low-resourced settings where bedside PD catheter insertion can be safely taught and is an acceptable dialysis modality, especially in settings where children with AKI would otherwise not survive.

**Graphical abstract:**

**Figure:**
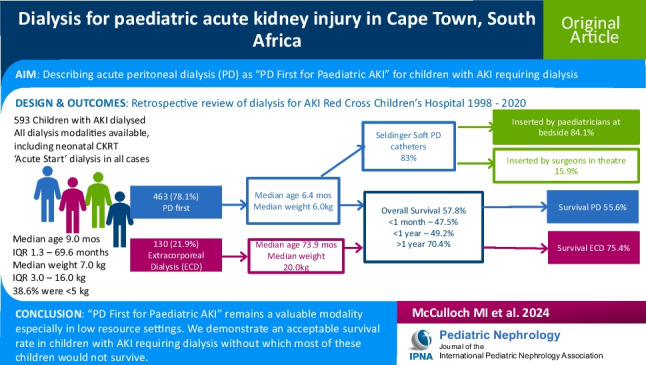
A higher resolution version of the Graphical abstract is available as Supplementary information

**Supplementary Information:**

The online version contains supplementary material available at 10.1007/s00467-024-06399-1.

## Introduction

Major advances in the management of acute kidney injury (AKI) globally, including improvements in kidney replacement therapies, have enabled even infants and small children to be dialysed [1]. However, most low and lower middle-income countries (LLMIC) still have very limited paediatric nephrology services and even more limited access to dialysis [2–10]. An international survey of AKI therapies showed that peritoneal dialysis (PD) is widely available [11]. Peritonitis remains a major concern but can be minimised with strict aseptic techniques even with locally produced PD fluids [12–15]. The use of haemodialysis (HD) or continuous kidney replacement therapy (CKRT), grouped together in this paper as extracorporeal dialysis (ECD), is more restricted than PD due to lack of trained paediatric staff, reduced availability of appropriate paediatric-sized equipment and consumables, limitations in numbers of machines, and cost. In addition, in children, acute HD may be challenging, particularly in small infants, in the absence of dedicated neonatal facilities. PD for AKI in children is therefore more feasible and more accessible globally and in lower resource settings is more scalable than HD, given less reliance on electricity, water, and machines.

Access to dialysis for AKI is lifesaving. In a recent systematic review from sub-Saharan Africa, mortality was 30–40% among children who received dialysis, compared with 74% among those who needed dialysis but did not receive it [8]. A variety of barriers precluded all children from accessing dialysis, including unaffordable out-of-pocket costs, erratic hospital resources, and late presentation. PD is the only possible dialysis modality for children in many settings where electricity and water supplies may be erratic and HD facilities do not exist. Supported by the International Pediatric Nephrology Association, the International Society of Peritoneal Dialysis, and the International Society of Nephrology, the “Saving Young Lives” (SYL) programme has permitted implementation of acute PD in various low resource settings [16, 17]. At times, despite lack of PD supplies, alternative catheters such as chest drains, urine catheters, and nasogastric tubes have been used, with PD fluid constituted at the bedside [18]. Lives have been saved, but these approaches should not be accepted as adequate. Much advocacy is required to improve access to dialysis for children with AKI in low resource settings.

In Cape Town, given cost considerations, we have instituted a “PD First” policy in all children with AKI requiring dialysis, meaning that PD is used as the initial modality of choice in all children without specific contraindications. This “PD first in Paediatric AKI” policy is distinct from the well-known “PD First” policy in adults, where PD is prioritized for chronic dialysis, given similar outcomes, scalability, and lower costs compared to chronic HD in some settings [19–21]. In adults, where resources permit, HD is generally preferred for AKI given its greater efficiency. Integrated into our “PD first for AKI” policy, PD catheters are often placed at the bedside by nephrologists, obviating the need for moving the patient to an operating theatre, dependence on a surgeon (who may not be available and may not be trained in laparoscopic techniques), avoiding delays in initiation of dialysis, and reducing costs. Acute/urgent-start dialysis can be commenced as soon as the PD catheter has been inserted [22, 23]. The “PD First in Paediatric AKI” approach has the added benefit of the possibility of implementation in small infants and children who may not otherwise have any access to dialysis [8, 9, 15].

The setting at the Red Cross War Memorial Children’s Hospital (RCWMCH) in Cape Town is relatively unique, in that we operate under significant resource limitations, but have access to the spectrum of acute dialysis options for children for all who require it. Given our “PD first” policy for AKI, we are uniquely placed to evaluate the outcomes of the policy in relation to outcomes in children receiving other forms of dialysis. These findings can inform policy development and advocacy for improved dialysis access for children elsewhere.

### Objectives

The study objectives were to describe the demographics of children who received dialysis for AKI over the study period, to describe the leading causes of AKI in our setting, and to evaluate the distribution of dialysis modalities used and methods of PD catheter insertion and the outcomes associated with these. Outcomes of interest included complications associated with PD catheter insertion, duration of dialysis, complications of dialysis, and survival on each dialysis modality for the cohort as a whole and stratified by weight and age.

## Methods

### Study design

Retrospective descriptive review of outcomes in all children aged birth to 18 years who received acute dialysis between 1998 and 2020 at RCWMCH, predominantly in the paediatric intensive care unit (PICU). Data were retrieved from a continuous paper-based database.

### Setting

The South African healthcare system comprises government-funded and private medical insurance services. Private health care is funded through insurance or out of pocket. The government funds medical care for South Africans without medical insurance at government institutions, with varying degrees of cost-sharing depending on an individual’s income. This includes acute and chronic dialysis and transplantation if individuals meet eligibility criteria. In addition, healthcare for children under 6 years of age is free.

The RCWMCH is a 300-bed, government-funded, tertiary-quaternary referral hospital that manages both government-funded patients and those with private medical insurance or private funding, as some highly specialised services are available only at RCWMCH and not in the private sector. In the kidney unit, the ratio of government to privately funded patients is 70:30, and all are treated equally. There is no selection bias in terms of children being turned down for treatment depending on parental affordability of treatment. This differs from some other LLMIC. Big geographic distances across a large country, however, may contribute to treatment bias for AKI depending on awareness and paediatric knowledge in district and local clinics.

The hospital primarily serves the needs of the greater Cape Town metropolitan region with roughly 1.1 million residents aged 0–14 years in 2021. The overall population increased by approximately 25% over the period of the study [24]. In addition, RCWMCH services a much bigger geographic region including the Western Cape province and the neighbouring Eastern and Northern Cape provinces (totalling 672,000 km^2^, more than 50% of South Africa). Finally, a small number of children are referred from other parts of southern Africa. In 2022, nearly 21 million, or 34% of South Africa’s total population of 62 million people, were children younger than 18 years. Although RCWMCH has a government-imposed paediatric cut-off of 13 years, some older children in chronic services still undergo treatment at the institution.

The hospital has a 22-bed PICU with approximately 1400 admissions per annum across all disciplines, including medical and surgical cases; the latter includes cardiothoracic and neonatal surgery. In addition, the hospital has a paediatric nephrology service that provides acute and long-term care for a wide variety of patients. Most patients undergo acute dialysis within the PICU in keeping with their overall condition, but some patients may also undergo acute dialysis within the high care area of the paediatric nephrology unit. Staffing in the PICU is a 1:1 nurse to patient ratio but may be a pair of senior and junior nurses per 2 beds as opposed to the high care unit where there is a 1:4 nurse to patient ratio. The full spectrum of dialysis modalities for both acute and chronic dialysis (including CKRT including the neonatal Carpe Diem® machine) and for the management of acute metabolic events is available. Most of the nursing staff are trained in PD management (specifically manual PD), but this is not the case for ECD for which there is only a small team of trained staff; ECD requires both nephrology dialysis staff and paediatric nephrology backup. However, our overarching unit policy is the “PD First in Paediatric AKI” approach, i.e. to consider PD (for acute and chronic dialysis) before other options and to use an “Acute/Urgent Start” approach, with PD catheters being inserted at the bedside by non-surgical nephrology or PICU staff [22, 23, 25, 26]. Note that for this study, we have excluded chronic dialysis as much as possible.

### Participants

#### Subject eligibility

Acute kidney injury (AKI) was defined according to KDIGO criteria as those with decreased urine output and/or rising creatine level. Infants and children requiring acute dialysis who were assessed to have a reasonable chance of recovering kidney function without long-term chronic kidney failure or severe disability were included in the study. In some cases, there may have been a suspicion of chronic kidney failure (KF), but if a child would have the option of transplantation, they were given a chance with acute PD and included in the analysis given that feasibility and complications of acute PD remain relevant.

Patients were excluded if deemed to have KF that would require long-term dialysis where transplantation was not deemed a possibility. Note that such patients undergo a multidisciplinary team review process to assist in determining eligibility for further dialysis and transplantation or palliative care as described elsewhere [27].

#### Clinical processes

Indications for dialysis included anuria/oliguria, acidosis, fluid overload, and hyperkalaemia and hyperphosphataemia not responding to medical therapy including trial of frusemide and aminophylline [28]. Inborn errors of metabolism, such as urea cycle defects with associated hyperammonaemia, are managed with PD as a temporising measure before moving to ECD, provided as either CKRT or HD. A variety of methods of insertion of dialysis catheters are described in Table 1.

**Table 1:** Methods of insertion of dialysis catheters

A. Peritoneal dialysis (PD) catheter insertion		
	**Infants/smaller children < 10 kg**	**Bigger children > 10 kg**
Preparation of prior to PD catheter insertion	• Placed under strict aseptic technique • Full theatre conditions including gowns and gloves • Ensuring urinary catheter in bladder and patent by flushing to prevent bladder perforation • Use of bedside ultrasound to assess bladder distension and increasingly identifying best PD catheter position • Bowel perforation is prevented by initial instillation of 20 mL/kg fluid into the peritoneal cavity via a vascular cannula, thus creating artificial ascites, prior to placing a PD catheter	
Specific PD catheter used	• Seldinger technique • Cook® PD (Straight 5Fr/8.5Fr) or Cook® Fuhrman drainage (pigtail 8.5Fr) • Uncuffed non-tunnelling	• Seldinger technique • “Peel-away” Tenckhoff catheters for bigger children • Uncuffed non-tunnelling
Operator medical	• Paediatric nephrologists/fellows/neonatologists	
Operator surgeon	• Rarely • Bedside in ICU in complicated cases • Small number of cases surgeons place cuffed PD catheters in operating theatre during cardiac or abdominal surgery	
At time of bedside PD catheter insertion	• Dose of vancomycin 10 mg/kg (or cefazolin 50 mg/kg) intravenously stat over 1 h slowly, unless already receiving antibiotics for their underlying condition • PD fluid is sent for microscopy and culture and, specifically, for a white cell count at the time of insertion and at any other time if concerns of peritonitis arise	
Infection surveillance	• Urine dipstick testing on the PD fluid is performed daily at the bedside to detect leucocytes • In cases of suspected peritonitis, dialysis effluent is sent for microbiological examination, and empiric intraperitoneal antibiotics (ceftazidime and vancomycin) are added as per local protocol until microbiological identification and sensitivities are available	
Trouble shooting tricks	• Flushing is attempted for blocked or poorly draining catheters • For leaking PD catheters, attempts are made to seal the leak with surgical glue or sutures • Pleural effusions related to PD are diagnosed on chest X-ray or on ultrasound. If identified, pleural fluid is aspirated and tested for glucose to confirm the presence of dialysis fluid. Treatment includes placing the patient head up at 30 degrees, reducing the fill volume of each dialysis cycle, and inserting an intercostal chest drain in cases where respiratory embarrassment occurs • In cases where PD fails or is not possible due to abdominal issues, it is changed to ECD either as HD or CKRT depending on stability of the patient	
B. Haemodialysis/extracorporeal dialysis catheter insertion		
	**Infants/smaller children < 10 kg**	**Bigger children > 10 kg**
Preparation of prior to PD catheter insertion	• Placed under strict aseptic technique • Use of ultrasound guided Seldinger technique • Dose of vancomycin 10 mg/kg (or cefazolin 50 mg/kg) intravenously stat over 1 h slowly, unless already receiving antibiotics for their underlying condition	
Access vessel used	• Preferably neck vessels either internal jugular vein (avoid subclavian vein) • Femoral as second option	
Specific HD catheter used	• Gamcath® 6.5Fr • Arrow® 5Fr (5 cm double lumen 18G and 20G (not a custom-made HD catheter) • For small infants—have used in less than 2 kg	Bigger children included sizes 7, 8, and 9 Fr (Medcomp® or Arrow®)
Operator medical	• Paediatric intensivists/anaesthetists/nephrologists • Neonatologists	• Paediatric intensivist/anaesthetists/nephrologists
Trouble shooting tricks	Heparin lock/other forms of locking, e.g. TPA	

Dialysis consumables were purchased from the two main companies servicing our facilities, i.e. Fresenius and Adcock (Baxter). Specific supplies and equipment such as infant and neonatal lines often require importation from Europe and North America, with a lengthy lead time for orders; this has become easier in recent years. Dialysis machines used over the study period are listed in Supplementary Table 1.

#### Data sources

The clinical team initiating dialysis was responsible for clinical data entry into a paper-based record, including age, weight, clinical indication for dialysis, type of dialysis used, type of dialysis catheter inserted including operator and location of insertion, duration of dialysis, complications, and patient outcomes. Paediatric-trained dialysis technologists recorded technical details of lines, dialysis times, and machine issues in the same database. This database was cross referenced with the PICU electronic patient database which also includes dialysis information.

#### Study size

Data of 593 children dialysed for acute AKI since 1998 were used in this study. Data were extracted from the database and were stratified into time quartiles according to dates, i.e. 1998–2003, 2004–2009, 2010–2015, and 2016–2020, to highlight changes that occurred over time including access to improved child-friendly dialysis equipment through the acquisition of modern Fresenius machines (HD Fresenius 5008 Paeds 2014, Fresenius CKRT Multifiltrate machine 2012) and Carpe Diem® neonatal CKRT machine in 2019 as well as improved dialysis catheter technology. This information is included in tables in a supplementary section.

#### Statistical methods

The record keeping was paper-based and retrospective, with approximately 10% missing data. Missing data were not imputed. Normally and non-normally distributed data were described and analysed using means and standard deviations or medians and interquartile ranges (IQR), respectively, with chi-squared, Wilcoxon sum rank, and Kruskal–Wallis tests used for comparisons, as appropriate. Analysis was performed using Stata Statistical Software: Release 17. College Station, TX, StataCorp LLC, and Microsoft Excel.

### Ethics

This study complied with the ethical guidelines and principles of the Helsinki Declaration of 2008, South African Guidelines for Good Clinical Practice, and the MRC Ethical Guidelines for Research and was approved by the Human Research Ethics committee of the University of Cape Town (Ethics approval UCT HREC Ref: 646/2015 [exp 28/2/2024]).

## Results

### Participants

Over the study period, 593 children (55.0% male) with AKI received dialysis with 463 (78.1%) receiving PD as the first dialysis modality (Table 2). The median age was 9.0 (range 0.03–219.3; IQR 1.3–69.6) months; most children (57.6%) were under 1 year old. The median age for those who received PD only was 6.4 months (IQR 0.9–30.4) vs. 73.9 months (IQR 17.5–113.9) for those who received ECD only (*p* < 0.001).

**Table 2:** Demographic and clinical details of patients requiring dialysis for acute kidney injury (AKI)

	*N*	Peritoneal dialysis	Extracorporeal dialysis	*p* Value
Male, *n* (%)	549	242 (55.3)	63 (56.8)	0.78
Age in months, median (IQR)	528	6.4 (0.92–30.4)	73.9 (17.5–133.9)	< 0.001
Neonate, *n* (%)	134	126 (27.6)	8 (8.0)	< 0.001
Infant, *n* (%)	182	166 (36.3)	16 (16.0)	
Child, *n* (%)	241	165 (36.1)	76 (76.0)	
Weight in kg, median (IQR)	502	6.0 (3.0–11.8)	20.0 (11.0–30.0)	< 0.001
Weight < 5 kg, *n* (%)	201	189 (46.0)	12 (11.0)	< 0.001
Weight 5–9.9 kg, *n* (%)	106	95 (23.1)	11 (10.1)	
Weight 10–19.9 kg, *n* (%)	98	69 (16.8)	29 (26.6)	
Weight 20–29.9 kg, *n* (%)	52	31 (7.5)	21 (19.3)	
Weight 30–39.9 kg, *n* (%)	42	17 (4.1)	25 (22.9)	
Weight > 40 kg, *n* (%)	21	10 (2.4)	11 (10.1)	
Underlying clinical condition resulting in AKI				
Burns		6 (1.3)	1 (1.1)	0.98
Cardiac medical^a^ and surgical		137 (29.8)	5 (5.4)	0.23
Encephalitis/meningitis		4 (0.9)	0	
Necrotising enterocolitis		22 (4.8)	0	
GIT medical		8 (1.7)	0	
GIT surgical		10 (2.2)	5 (5.4)	0.74
Liver/metabolic		28 (6.1)	11 (12.0)	0.53
Haematology/oncology		16^b^ (3.5)	21^c^ (22.8)	0.09
Kidney transplant		0	5 (5.5)	
Kidney failure		24 (5.2)	17 (18.5)	0.17
Haemolytic uraemic syndrome		36 (7.8)	2 (2.2)	0.77
Other kidney		22 (4.8)	13 (14.1)	0.33
Respiratory		9 (2.0)	0	
Sepsis/shock^d^		122 (26.5)	6 (6.5)	0.27
Trauma		5 (1.1)	5 (5.4)	0.70
Miscellaneous		10 (2.2)	1 (1.1)	0.94
Survival	526	254 (55.6)	52 (75.4)	0.002

*GIT* gastrointestinal tract

^a^Nine had myocarditis

^b^Two had tumour lysis syndrome

^c^Ten had tumour lysis syndrome

^d^Nine had severe acute malnutrition with oedema

### Descriptive data

The weights of patients dialysed ranged from 0.9 to 62.0 kg (median 7.0 kg, IQR 3.0–16.0 kg), with 38.6% < 5 kg and 61.6% < 10 kg, consistent with the age ranges. More than half (53.5%) of all patients dialysed were under 1 year of age with a weight under 10 kg (58.6%). Overall, 69.1% of patients receiving PD weighed less than 10 kg, whereas 52.3% of children receiving HD weighed over 20 kg. From a clinical perspective, patient median weights remained relatively constant over the study period ranging from 6.0 to 10.8 kg (Supplementary Table 2).

## Main results

### Conditions associated with dialysis requiring AKI

The true prevalence of community vs. hospital acquired AKI cannot be ascertained from our data as these were all children admitted to PICU who required dialysis. The top five categories of conditions associated with AKI requiring dialysis in our study remained fairly constant over time (Supplementary Table 3): medical and surgical cardiac conditions (*n* = 145, 26.8%), primary kidney pathology (*n* = 113, 20.9%), sepsis/septic shock requiring inotropic support (*n* = 128, 24.4%), gastrointestinal conditions (*n* = 59, 10.9%), and oncological disorders (*n* = 30, 5.5%) (Table 2). Nine (1.6%) children had toxicity due to an administered toxin/traditional or other medication.

Children with burns or severe acute malnutrition with oedema had poor outcomes: 6 of 7 (85.7%) and 7 of 9 (77.0%) children with these respective conditions died. The most frequent indication for dialysis for kidney conditions was haemolytic uraemic syndrome (HUS) (*n* = 38, 33.6%), while 41 (36.3%) had underlying chronic kidney failure, and 9 (8.0%) had received a kidney transplant.

### Peritoneal dialysis modalities

Most children were commenced on dialysis in the PICU, although for 61 (10%) children, dialysis was initiated in the paediatric nephrology high care area while awaiting a bed in the PICU (Table 3). PD consisted predominantly of manual PD, which was used more commonly in infants under 1 year. Automated cycling PD was used in older children weighing > 5 kg (in view of 100 mL fill volumes) which reduced the nursing workload related to manual PD. Overall, most children (463 (78.1%) received only PD, with manual and automated PD being performed in 302 (65.2%) and 154 (33.3%) cases, respectively, with a small number receiving both.

**Table 3:** Mode of dialysis for paediatric AKI by study period, *n* (%)

Period	PD only			ECD only	PD and ECD	Total
	APD	MPD	APD + MPD			
1998–2003	46 (28.8)	110 (68.8)	0	2 (1.2)	3 (1.9)	161 (100)
2004–2009	62 (39.5)	72 (45.8)	2 (1.3)	18 (11.4)	3 (1.9)	157 (100)
2010–2015	28 (18/3)	82 (53.6)	4 (2.61)	37 (24.2)	1 (0.6)	153 (100)
2016–2020	18 (14.8)	38 (31.2)	1 (0.9)	57 (46.7)	8 (6.6)	122 (100)
Total	154 (26.0)	302 (50.9)	7 (1.2)	114 (19.2)	15 (2.5)	593 (100)

*PD* peritoneal dialysis, *ECD* extracorporeal dialysis, *APD* automated peritoneal dialysis, *MPD* manual peritoneal dialysis

### Peritoneal dialysis catheter types and methods of insertion

Most PD catheters (*n* = 480/578; 83%) were inserted at the bedside using a Seldinger technique. These included 335/578 (60.9%) Cook® catheters (straight PD catheters and pigtail Fuhrman catheters), 145/578 (26.3%) peel-away PD catheters, and 45/578 (8.2%) surgical Tenckhoff PD catheters. Paediatric nephrologists/intensivists inserted 234 (47.8%) PD catheters, and trainee nephrology fellows inserted 178 (36.3%) PD catheters under supervision as an essential part of their training. Surgeons inserted PD catheters in 78 (15.9%) cases either at the bedside or in the operating theatre.

### Complications of peritoneal dialysis

The overall complication rate was 99/560 (17.7%) and included mainly technical problems including blockage or poor drainage (*n* = 72/560, 12.9%), leakage (*n* = 5, 3.9%), displacement of the PD catheter (*n* = 6, 4.7%), pleural effusion secondary to PD (*n* = 4, 3.2%), fungal peritonitis requiring discontinuation (*n* = 3, 2.4%), and bladder perforation (*n* = 2, 1.6%) not requiring surgical intervention. There were no cases of bacterial peritonitis documented in this study. No bowel perforations were noted, but discoloured ascites on insertion alerted us to the presence of necrotic bowel or peritonitis requiring change in management in 7 (5.5%) cases. Where PD did not function adequately, ECD was used (*n* = 17, 2.9%).

### Extra-corporeal dialysis (including HD and CKRT) for AKI

For 130 (21.9%) children, only ECD was used. For 17 (2.9%) children, both PD and ECD modalities were used; this occurred if one modality was unable to provide adequate dialysis or if PD was not functioning optimally in terms of fluid removal (Table 3). There was a trend toward increasing use of ECD over time. Of the 130 children who received ECD with or without PD, the type of ECD used was recorded for 115, as follows: CKRT 47 (36.2%), Carpe Diem CKRT Neonates 9 (6.9%), CKRT and HD 31 (23.8%), and HD only 28 (21.5%).

In the first 2 quartiles, only 2 (1.2%, 1998–2003) and 18 (11.4%, 2004–2009) HD catheters were used, compared with 37 (24.2%, 2010–2015) and 57 (46.7%, 2016–2020) HD catheters used in the last 2 quartiles studied.

### Duration of dialysis

The duration of dialysis for AKI was < 5 days in 337 cases (56.8%), 5–7 days in 70 cases (11.8%), 8–14 days in 50 (8.4%) cases, > 14 days in 47 cases (7.9%), and was not documented in 89 (15.1%) cases (Table 4).

**Table 4:** Survival proportions by dialysis modality and duration in paediatric patients with AKI

	Survival proportions, *n* (%)		
Duration of dialysis	Peritoneal dialysis	Extracorporeal dialysis	*p* Value
≤ 1 day	34 (28.8)	6 (50.0)	0.166
2–7 days	122 (60.4)	21 (72.4)	0.295
8–14 days	25 (67.6)	3 (60.0)	0.79
> 14 days	13 (59.1)	15 (93.8)	0.028

Most children (*n* = 407, 80.8%) were dialysed for 7 days or less. While a set lifespan for the use of acute PD catheters was not defined, we continued to use the inserted PD catheter for longer than 7 days in ~ 10% of children if the catheter remained functional, while we remained vigilant for infection.

### Outcome data

#### Survival of patients with AKI requiring dialysis

Overall, 314 (57.8%) children survived. The median age of those who survived was 11.2 (1.8–80.9) months; those who died had a median age of 4.8 (0.7–15.3) months (*p* < 0.0001). Neonates and infants had poorer survival (47.5% and 49.2%, respectively) compared with older children (70.4%, *p* < 0.0001). Similarly, survival was worse in the lowest weight category (< 5 kg, 47.6%) and higher in the patients over 40 kg (70.0%, *p* < 0.0001) (Table 5).

**Table 5:** Survival proportions by dialysis modality and patient weight in paediatric patients with AKI

	Survival proportion, *n* (%)		
Weight	Peritoneal dialysis	Extracorporeal dialysis	*p* Value
< 5 kg	88 (46.6)	7 (70.0)	0.23
5–9.9 kg	48 (50.5)	4 (57.1)	0.80
10–19.9 kg	43 (62.3)	10 (76.9)	0.38
20–29.9 kg	20 (66.7)	10 (76.9)	0.56
30–39.9 kg	15 (88.2)	13 (86.7)	0.90
> 40 kg	7 (70.0)	7 (77.8)	0.74
Total	221 (54.3)	51 (76.1)	0.003

Survival was significantly greater in the group who received ECD only compared to those who received PD only (55.6% vs. 75.4%, *p* = 0.002). Survival remained relatively stable over time and by modality (Supplemental Table 4). Five children with AKI developed chronic kidney disease.

#### Paediatric index of mortality (PIM) scoring in PICU

Most children in this study were critically ill with unfavourable PIM scores as an indicator of the severity of their illness. In view of the lengthy period of the study (1998–2020), comparing actual PIM scoring across the whole cohort was not possible given the changing PIM scoring systems during the study period. Of those with a recorded PIMS score (*n* = 327), predicted mortality rates were around 10–12%.

## Discussion

We present a large series of children with AKI managed predominantly with “PD First for Paediatric AKI” as the main form of dialysis over a 20-year period in a middle-income African country. This cumulative experience has allowed us to become trailblazers in this field, especially with paediatric nephrologists gaining experience in the bedside-based insertion of soft PD catheters inserted using the Seldinger technique. Elements identified as important in a successful “PD First in Paediatric AKI program” include the following: nephrologist experience and expertise, PD catheter access, and psychosocial support for PD patients (especially for those subsequently requiring chronic PD) [29]. It is important to recognise that RCWMCH is a tertiary-quaternary hospital, and, thus, we have had increasing access over time to use of other dialysis modalities when PD was not clinically possible, including the Carpe Diem Neonatal CKRT machine. We therefore highlight our experiences with improved techniques of dialysis, showing that this transition may be possible in other lower resource settings in the coming years.

At RCWMCH, half of all 1400 annual PICU admissions are for infants < 1 year of age. The dialysis numbers for AKI reflect this in that more than half of the patients who received dialysis were under 1 year of age with a weight < 10 kg. PD was used predominantly in infants compared to ECD used in older children. A quarter of patients requiring dialysis were children with cardiac conditions, which mirrors high income settings as reflected in the AWARE and AWAKEN studies [30–34]. Primary kidney pathology (predominantly HUS) was a common condition associated with AKI in our setting. The requirement for dialysis in sepsis/shock appears to have declined over time (Supplementary Table 3), which may be due to earlier recognition and appropriate treatment of sepsis/shock or in its reduction in part due to expanded immunisation schedule and/or improved socioeconomic conditions. Oncology patients despite a reduction in tumour lysis syndrome with the use of rasburicase still account for a number of dialysis cases, due to an increasing stem cell transplant population who then present with sepsis.

Peritoneal dialysis remains our first line dialysis therapy unless there are extreme surgical abdominal issues such as an open postoperative abdomen. As we employ a “PD First in paediatric AKI” program that uses an “Acute/Urgent start” approach, our nurses are PD-trained in keeping with many centres in the world, where staff are more comfortable with providing PD than ECD [11, 18, 35]. The modalities of PD included manual PD predominantly for infants under 1 year and automated cycling PD in older children. In manual PD, a group of infants received continuous flow PD with improved clearances; therefore, future plans will include this modality in training for LMIC [36]. The use of automated cycling PD machines for children requiring dialysis for AKI (compared with chronic PD) is also a novel way of relieving staff pressures in the PICU. Locally produced PD fluid is a safe alternative to commercial PD fluid for regions where commercial fluid is unavailable [12–15]. PD is especially suited to neonates and infants where venous access is challenging, as well as for postoperative cardiac patients where surgeons may place PD catheters at the end of cardiac surgical procedures [37].

Catheters used for PD were mainly Seldinger Cook® or Peelaway catheters inserted at the bedside by the paediatric team (nephrologists/intensivists or fellows under supervision), with surgical backup needed in a minority of mainly complex cases. Custom made Cook® straight PD catheters have been discontinued, and we have successfully used Fuhrman Cook® Pigtail catheters as an alternative. Technical problems related to catheter leakage, blockage, or dislodgement were managed practically with surgical glue to leaking catheter sites and re-wiring/replacement of PD catheters at the bedside. This study also highlights a training opportunity for paediatric nephrology fellows, teaching them to insert PD catheters under the supervision of paediatric nephrology consultants; relatively few complications were documented if the protocol was followed. This also allowed for catheter training in cases requiring continuous flow PD [36]. As seen in adult studies, there did not appear to be any safety concerns with non-surgeon nephrologists placing PD catheters at the bedside, provided that existing protocols were followed, specifically urethral catheter placement prior to PD catheter insertion [15].

Peritonitis rates were very low, likely due to attention to detail in providing strict aseptic technique at the bedside—“bringing theatre to the bedside”. In addition, many patients were already on systemic antibiotics for the underlying conditions that led to AKI. In rare cases where peritonitis was diagnosed, this was treated with intraperitoneal antibiotics without removal of the acute PD catheters, in the same way as endotracheal tubes are not removed in the diagnosis of ventilator-associated pneumonia. The exception was for fungal peritonitis episodes where acute catheters were removed, and systemic antifungal treatment initiated.

The use of PD declined over the duration of the study (*p* for trend = 0.08) from 96.9% in the first quartile to 46.7% in the last quartile. As mentioned previously, this likely reflects improved availability of access to ECD machines in our setting, especially for neonates through the acquisition of the Carpe Diem® machine. Neonates referred for dialysis may also have had more abdominal pathology, precluding the use of PD [38]. These neonates would previously have died without the benefit of a trial of dialysis. Consequently, significant in-service training was required for the medical teams (doctors, technologists, and nurses) comfortable with PD but requiring upskilling in the provision of ECD, especially for smaller children. Additionally, HD catheters that are more suitable for infants are now available. Experience with ECD has grown, but with resultant cost implications [39–41].

Overall, the mortality rate in our study for paediatric AKI patients requiring dialysis was 41% which is comparable to other LMIC centres such as reported in Thailand (41.5%) [42] and India (36.8%) [43]. The mortality observed here is like that reported in children who received dialysis in the systematic review of dialysis requiring AKI in sub-Saharan Africa (30%) [8, 44]. Similar mortalities have been reported in critically ill children in North America treated with CKRT [45, 46]. In the AWARE study, the specific mortality of patients receiving dialysis was not reported, but the use of kidney replacement therapy was a strong predictor of death in children with severe AKI (odds ratio, 3.38; 95% CI, 1.74–6.54) [47]. Relevant here, however, may be that children in LMIC often present much later for medical care and that children in higher income countries may have greater degrees of complexity and multimorbidity. Therefore, it is difficult to compare outcomes. In low resource settings small infants may not be dialysed or children might only be dialysed for a limited time, such as 5 days, if parents have to pay for this out-of-pocket; these factors likely impact mortality rates [48].

Encouragingly, in our study, there has been an overall trend toward improved survival (56.7 to 62.8%; Supplementary Table 4) over the last 5 years. Multiple reasons may underlie this observation, including earlier referral and commencement of dialysis, as well as the availability of ECD for small infants that was not previously available. On review of the survival per modality, PD carried a 44.4% mortality compared with mortality of 24.6% on ECD (*p* = 0.002). This may reflect better outcomes in the older patients for whom CKRT was possible.

In our study, lower survival was seen in neonates and infants under 1 year of age compared to older children (47.5% vs. 70.4%, *p* < 0.0001; Supplementary Table 5) as well as those in the lower weight categories (47.6% vs. patients over 40 kg, 70.0%, *p* < 0.001; Table 5). These younger and smaller children were predominantly dialysed with acute PD. This may suggest a higher mortality on PD, but these findings may also reflect that these younger and smaller patients were sicker and were historically more difficult to dialyse due to lack of availability of all forms of dialysis equipment for small babies. Given these potential confounders, we cannot clearly determine whether PD is inferior to ECD in our setting given that PD, being more accessible and affordable, is used first. Future studies need to be designed to understand whether some subsets of patients may indeed do better with ECD.

### Limitations

Our study has several limitations, including the paper-based record keeping with approximately 10% missing or incomplete data. In addition, despite our cohort being large by paediatric standards, it remains a single centre study from a less well-resourced region and thus has limited generalizability. PIM and AKI scoring systems, such as the Kidney Disease: Improving Global Outcomes (KDIGO) score, were not able to be applied retrospectively. We do not currently have a PICU follow-up clinic, but some of these patients do filter through to the kidney clinic for follow-up. The long-term outcomes of the children reported here therefore remain unknown.

### Strengths

This study does have significant strengths, in that it has similar numbers to that of Abdelraheem from Sudan [35] and is one of the first of its kind to report a 20-year review of dialysis of children in Africa. The data collection was largely complete and included details on catheter types and dialysis techniques which are infrequently reported. Specifically, we analysed trends over time and found that the use of “PD First for paediatric AKI”, with soft PD catheters inserted with Seldinger technique, by nephrologists/trainees under supervision at the patient’s bedside, is safe, feasible, and successful and can be conducted with simple infrastructure. This should be encouraging to colleagues in similar settings who are beginning to scale up acute dialysis for children with AKI. When done carefully, PD does not need to be considered second best. Cost analysis of PD vs*.* ECD is beyond the scope of this study and will be reported on separately.

### Recommendations

Following our study, we would like to make some recommendations which may be beneficial in settings where paediatric AKI is managed:

- Starting a “PD First program for Paediatric AKI” is lifesaving even in the absence of commercially produced PD catheters, fluids and formal surgical expertise.
- Acute PD can be applied in a wide size range of paediatric patients including small neonates.
- Perform Acute Start PD at the bedside using Seldinger inserted soft drainage catheters or peelaway Tenckhoff catheters with immediate commencement of PD.
- With custom made Cook® straight PD catheters being taken off the market, Cook® Pigtail (Fuhrman) catheters, although not officially licensed for PD, can been used safely and successfully.
- Perform bedside ultrasound at time of PD catheter insertions to ensure empty bladder and identify bowel.
- Non-surgically trained personnel including paediatric nephrologists/intensivists can insert PD catheters at the bedside in appropriate patients if trained correctly.
- The duration of PD with catheters inserted at the bedside can exceed 7 days provided sterile techniques are adhered to, and there is infection surveillance (Supplementary Table 6).
- As PD programs develop, encourage advocacy for access to more advanced ECD dialysis facilities for patients where PD may not be possible.
- Establish an electronic data collection system for an “PD First for Paediatric AKI” program.

Evaluation of the economics of PD vs. HD/CKRT is urgently required. Long-term follow-up clinics for graduates of AKI therapy are also required but need appropriate manpower. In 2013, the International Society of Nephrology launched the “0 by 25” campaign with the goal that no patient should die from preventable or untreated AKI in low-resource areas by 2025 [49]. This paper aims to assist colleagues in other LMIC to achieve this goal, even in the absence of expensive equipment and consumables.

## Conclusion

“PD First for Paediatric AKI” remains a valuable acute/urgent start modality for management of AKI in children, especially in less well-resourced regions where it is possible to safely teach the Seldinger technique of insertion of PD catheters at the bedside using pigtail catheters or peel away technology. Internationally, even in well-resourced countries, with the discontinuation of custom-made Cook® straight PD catheters, we have shown that Cook® Pigtail (Fuhrman) catheters can be used successfully; this may solve the current shortage crisis. As PICU and/or high care units become more established in less well-resourced regions and access to equipment for small children (including HD catheters and paediatric CKRT machines) becomes increasingly available, practice does change. However, this should not prevent those in regions who do not have access to this kind of equipment from using PD, as it has shown an acceptable survival rate in children with AKI. Rather than attempting no intervention in children with AKI when ECD is not available, providing PD acutely using improvised equipment and PD fluid is both possible and lifesaving.

## Supplementary Information

Below is the link to the electronic supplementary material.

Graphical abstract (PPTX 81 KB)

Supplementary file2 (DOCX 57 KB)

Supplementary file3 (PDF 25 KB)

## Data Availability

The datasets generated during and/or analysed during the current study are available from the corresponding author on reasonable request.
