# Correction: Dialysis for paediatric acute kidney injury in Cape Town, South Africa

**DOI:** 10.1007/s00467-025-06880-5

**Published:** 2025-09-09

**Authors:** Mignon I. McCulloch, Valerie A. Luyckx, Brenda Morrow, Peter Nourse, Ashton Coetzee, Deveshni Reddy, Christel Du Buisson, Jonathan Buckley, Ilana Webber, Alp Numanoglu, Gina Sinclair, Candice Nelson, Shamiel Salie, Kirsten Reichmuth, Andrew C. Argent

**Affiliations:** 1 Red Cross War Memorial Children’s Hospital (RCWMCH), Rondebosch, Cape Town, South Africa; 2 University of Cape Town, Cape Town, South Africa; 3 Tygerberg Children’s Hospital, University of Stellenbosch, Stellenbosch, South Africa

**Correction: Pediatric Nephrology (2024) 39:2807-2818**

10.1007/s00467-024-06399-1

The original online version of this article has been revised. Complications occurred in 99/560 (17.7%) children receiving PD. The calculation for blocked PD catheters on page 2812 was originally quoted as:

“The overall complication rate was 127/560 (22.7%) and included mainly technical problems including blockage or poor drainage (*n* = 72, 56.7%)…”. However, this should have read: (*n* = 72/560 – 12.9%)."

The original article has been corrected.

