# Supplementary material for: Dialysis for paediatric acute kidney injury in Cape Town, South Africa
Source: Pediatr Nephrol. 2024 May 11;39(9):2807–18. doi: 10.1007/s00467-024-06399-1 (PMC11272748; doi:10.1007/s00467-024-06399-1)
Supplement: Supplementary file 1 — Graphical abstract (PPTX 81 KB) [file 467_2024_6399_MOESM1_ESM.pptx]

## Slide 1
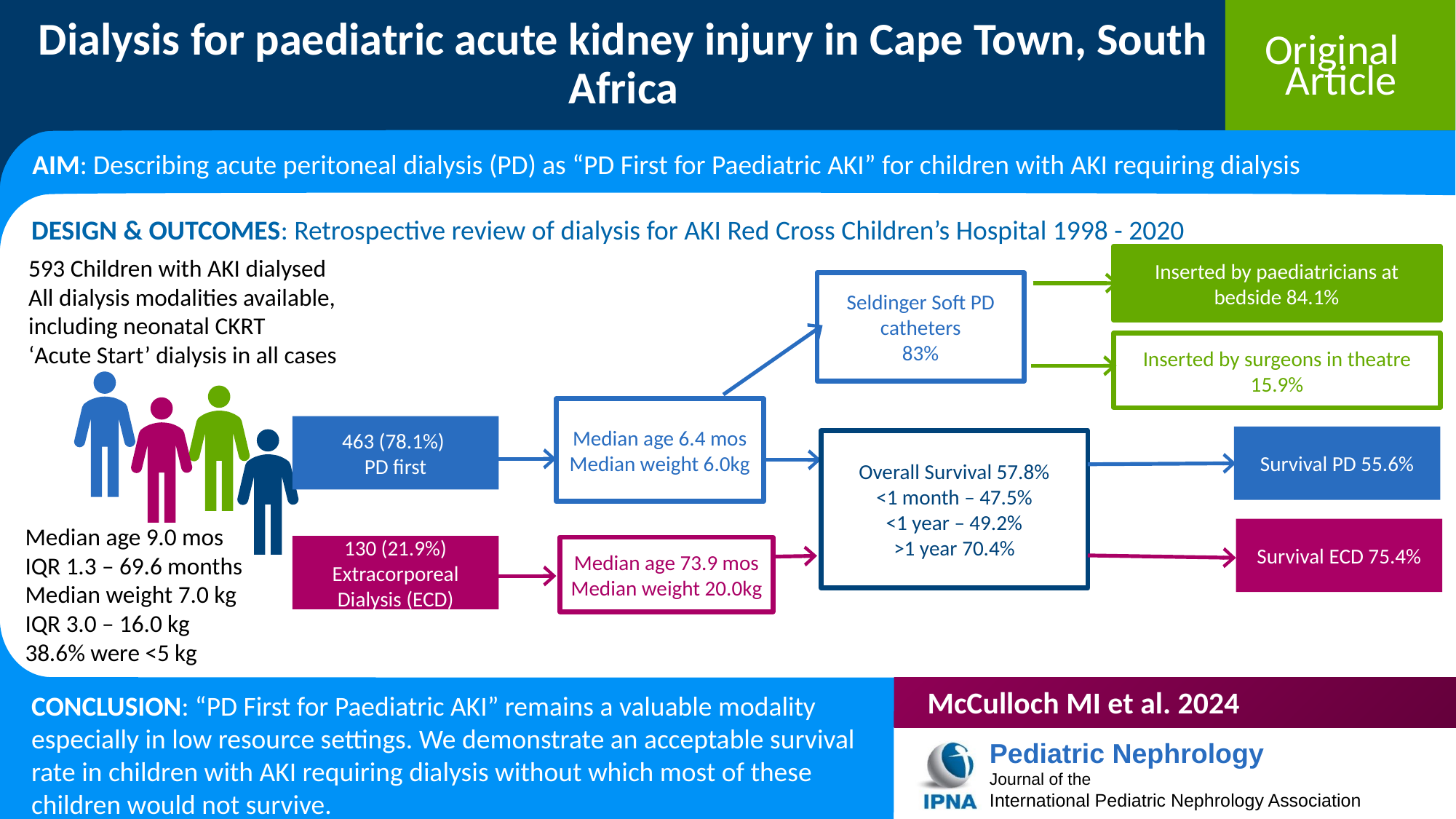

Dialysis for paediatric acute kidney injury in Cape Town, South Africa
AIM: Describing acute peritoneal dialysis (PD) as “PD First for Paediatric AKI” for children with AKI requiring dialysis
DESIGN & OUTCOMES: Retrospective review of dialysis for AKI Red Cross Children’s Hospital 1998 - 2020
Inserted by paediatricians at bedside 84.1%
593 Children with AKI dialysed
All dialysis modalities available,
including neonatal CKRT
‘Acute Start’ dialysis in all cases
Seldinger Soft PD catheters
83%
Inserted by surgeons in theatre 15.9%
Median age 6.4 mos
Median weight 6.0kg
463 (78.1%)
PD first
Survival PD 55.6%
Overall Survival 57.8%
<1 month – 47.5%
<1 year – 49.2%
>1 year 70.4%
Median age 9.0 mos
IQR 1.3 – 69.6 months
Median weight 7.0 kg
IQR 3.0 – 16.0 kg
38.6% were <5 kg
Survival ECD 75.4%
130 (21.9%)
Extracorporeal Dialysis (ECD)
Median age 73.9 mos
Median weight 20.0kg
McCulloch MI et al. 2024
CONCLUSION: “PD First for Paediatric AKI” remains a valuable modality especially in low resource settings. We demonstrate an acceptable survival rate in children with AKI requiring dialysis without which most of these children would not survive.
