# Supplementary material for: Dialysis for paediatric acute kidney injury in Cape Town, South Africa
Source: Pediatr Nephrol. 2024 May 11;39(9):2807–18. doi: 10.1007/s00467-024-06399-1 (PMC11272748; doi:10.1007/s00467-024-06399-1)
Supplement: Supplementary file 2 — Supplementary file2 (DOCX 57 KB) [file 467_2024_6399_MOESM2_ESM.docx]

| Supplementary Table 1. Dialysis machines used for AKI over the study period | | | |
| --- | --- | --- | --- |
| **Peritoneal Dialysis** | | **Extracorporeal Dialysis** | |
| Manual | Automated | Haemodialysis | CRRT |
| Improvised Baxter products  1996–2010 | PacXtra  1998–2002 | Fresenius 4008B  2001–present  Fresenius 4008S  2008–present | Gambro AK10  1999–2003  Baxter BM25  2004–2012 |
| Fresenius PD Paeds system, 2010 | Homechoice  2002–2018 | Fresenius 5008 Paeds 2014–present | Fresenius Multifiltrate 2012–present |
| CFPD Automated BM25/manual  2008–2014 | Claria  2018–present |  | Carpe Diem  2019–present |
| PD, peritoneal dialysis; CFPD, Continuous Flow Peritoneal Dialysis, CRRT, Continuous Renal Replacement Therapy | | | |

| **Supplementary Table 2**. **Demographic details of patients requiring dialysis for AKI, stratified by study period** | | | | | | |
| --- | --- | --- | --- | --- | --- | --- |
|  | **N** | **1998–2003** | **2004–2009** | **2010–2015** | **2016–2020** | **p-value** |
| Sex, male | 522 | 83 (53.9) | 70 (51.9) | 79 (61.2) | 54 (51.9) | 0.387 |
| Age in months, median (IQR) | 502 | 6.6 (1.1–38.2) | 9.1 (1.2–34.2) | 5.3 (0.8–45.3) | 13.1 (2.6–112.3) | 0.009 |
| Neonate, n (%) | 140 | 37 (23.6) | 35 (25.6) | 44 (32.8) | 24 (20.9) | 0.080 |
| Infant, n (%) | 177 | 60 (38.2) | 44 (32.1) | 42 (31.3) | 31 (26.9) |  |
| Child, n (%) | 226 | 60 (38.2) | 58 (42.3) | 48 (35.8) | 60 (52.2) |  |
| Weight in kg, median (IQR) | 475 | 6.0 (2.6–12.0) | 7.0 (3.3–12.8) | 6.0 (3.0–13.0) | 10.8 (4.0–28.0) | <0.001 |
| <5kg, n (%) | 195 | 57 (47.9) | 47 (35.6) | 60 (48.0) | 31 (29.3) | 0.001 |
| 5–9.9kg, n (%) | 102 | 20 (16.8) | 42 (31.8) | 23 (18.4) | 17 (16.0) |  |
| 10–19.9kg, n (%) | 85 | 23 (19.3) | 23 (17.4) | 19 (15.2) | 20 (18.9) |  |
| 20–29.9kg, n (%) | 45 | 6 (5.0) | 11 (8.3) | 13 (10.4) | 14 (13.2) |  |
| 30–39.9kg, n (%) | 35 | 9 (7.6) | 4 (3.8) | 7 (5.6) | 9 (13.9) |  |
| >40kg, n (%) | 20 | 4 (3.4) | 4 (3.0) | 3 (2.4) | 9 (8.5) |  |

| **Supplementary Table 3. Underlying conditions of paediatric patients leading to AKI requiring dialysis, stratified by study period** | | | | | |
| --- | --- | --- | --- | --- | --- |
|  | **1998-2003** | **2004-2009** | **2010-2015** | **2016-2020** | **Total** |
| Burns | 2 (1.3) | 2 (1.5) | 3 (2.3) | 0 | 7 (1.3) |
| Cardiac | 35 (22.0) | 48 (33.6) | 36 (25.7) | 26 (23.3) | 145 (26.2) |
| - medical | 14 (40.0) | 10 (20.8) | 10 (27.7) | 3 (11.5) |  |
| - surgical | 21 (60.0) | 38 (70.2) | 26 (72.2) | 23 (88.5) |  |
| Encephalitis/meningitis | 2 (1.2) | 2 (1.4) | 0 | 0 | 4 (0.7) |
| GIT NEC | 12 (7.5) | 2 (1.4) | 7 (4.9) | 1 (0.9) | 22 (4.1) |
| GIT medical | 11 (6.9) | 6 (4.4) | 2 (1.5) | 1 (0.9) | 20 (3.7) |
| GIT surgical | 5 (3.1) | 2 (1.5) | 3 (2.3) | 7 (6.3) | 17 (3.1) |
| Liver | 2 (1.2) | 5 (3.7) | 3 (2.3) | 3 (2.7) | 13 (2.4) |
| Metabolic | 7 (4.4) | 10 (7.3) | 6 (4.5) | 5 (4.5) | 28 (5.2) |
| Oncological | 9 (5.6) | 5 (3.5) | 11 (7.6) | 10 (8.6) | 35 (6.2) |
| - tumour lysis syndrome | 4 (44.5) | 3 (60.0) | 7 (63.6) | 3 (30.0) |  |
| Other | 2 (1.2) | 0 | 0 | 2 (1.7) | 4 (0.7) |
| Kidney | 31 (19.2) | 25 (17.5) | 26 (18.1) | 39 (33.6) | 121 (21.5) |
| - kidney transplant | 0 | 1 (4.0) | 2 (7.7) | 6 (15.4) |  |
| - kidney failure | 0 | 4 (16.0) | 3 (11.5) | 6 (15.4) |  |
| - HUS | 10 (32.2) | 12 (48.0) | 9 (36.0) | 8 (20.5) |  |
| Respiratory | 1 (0.6) | 2 (1.4) | 5 (3.5) | 1 (0.9) | 9 (1.6) |
| Rheumatology | 1 (0.6) | 1 (0.7) | 2 (1.4) | 1 (0.9) | 5 (0.9) |
| Sepsis | 36 (22.6) | 20 (14.0) | 30 (20.8) | 15 (12.9) | 101 (17.9) |
| Shock | 10 (6.2) | 13 (9.1) | 6 (4.2) | 0 | 29 (5.1) |
| Trauma | 4 (2.5) | 1 (0.7) | 3 (2.1) | 2 (1.7) | 10 (1.8) |
| Total | 161 (100) | 143 (100) | 144 (100) | 116 (100) | 570 (100) |
| AKI, acute kidney injury; GIT, gastrointestinal tract; NEC necrotising enterocolitis; HUS, haemolytic uraemic syndrome | | | | | |

| **Supplementary Table 4 Survival of paediatric AKI patients per modality over time** | | | | | |
| --- | --- | --- | --- | --- | --- |
|  | **Survival, n (%)** | | | | |
| **Period** | **PD only** | **ECD only** | **PD and ECD** | **p**-**value** | **Total** |
| **1998–2003** | 89 (57.4) | - | 1 (25.0) | 0.17 | 90 (56.6) |
| **2004–2009** | 74 (55.6) | 1 (100) | 2 (67.7) | 0.63 | 77 (56.2) |
| **2010–2015** | 61 (54.0) | 14 (73.7) | 1 (50.0) | 0.27 | 76 (56.7) |
| **2016–2020** | 30 (53.5) | 37 (75.5) | 4 (50.0) | 0.05 | 71 (62.8) |
| **Total** | 254 (55.6) | 52 (75.4) | 8 (47.0) |  | 314 (57.8) |
|  |  |  |  |  | p for trend =0.73 |
|  | PD, peritoneal dialysis; ECD, extracorporeal dialysis | | | | |

**Supplementary Table 5 Age of paediatric AKI patient receiving dialysis by survival**

| **Age** | **Alive** | **Died** | **Total** |
| --- | --- | --- | --- |
| **Child (> 1 year)** | 157 (70.4) | 66 (29.6) | 223 (100) |
| **Infant (<1 year)** | 88 (49.2) | 89 (50.3) | 177 (100) |
| **Neonate (<1 month)** | 66 (47.5) | 73 (52.5) | 139 (100) |
| **Total** | 311 (57.7) | 228 42.3) | 539 (100) |

**p<0.0001**

**Supplementary Table 6 Time on dialysis**

| **Time on dialysis** | **Number of cases** |
| --- | --- |
| Total | 351(100%) |
| 2-4days | 185(52.7%)* |
| 5-7days | 70(19.9%)* |
| 8-14days | 49(14.0%) |
| >14days | 47(13.4%) |

*Majority of patients(255;72.6%) required dialysis <7 days overall
